# Primary hydatid cyst of the pancreas with a hepatic pedicule compression

**DOI:** 10.1186/1757-1626-2-201

**Published:** 2009-11-18

**Authors:** Abdelmalek Ousadden, Hicham Elbouhaddouti, Karim H Ibnmajdoub, Khalid Mazaz, Khalid AitTaleb

**Affiliations:** 1 Service de chirurgie viscérale, Hôpital des spécialités, CHU de Fès, Route de Sidi Harazem, Fès, 30070, Morocco

## Abstract

**Introduction:**

Primary pancreatic hydatid cyst is extremely rare and may be a causative factor for obstructive jaundice.

**Case presentation:**

A 27-year-old woman presented with obstructive jaundice, vomiting, pruritus, abdominal pain and an epigastric mass. A diagnosis of a pancreatic cyst causing a compression of the common bile buct was established by ultrasonography and CT scan before surgery. Hydatic serology was negative. The treatment consisted of the resection of the protruding dome with a drainage of the residual cavity and an omentoplasty. The recovery was uneventful and the patient has remained symptom free so far.

**Conclusion:**

The primary hydatid cyst of the pancreas may be a causative factor for obstructive jaundice and should be considered in the differential diagnosis of all cystic masses in the pancreas, especially in endemic areas.

## Introduction

Hydatid cysts are extremly rarely found in the pancreas. Even in the endemic countries, its incidence varies from 0.14 to 2% as compared to the other sites of hydatid disease [1-8]. For us, the incidence is about one case for 300.

The head of the pancreas is the most frequent location (57%), followed by the corpus (24%) than the tail (19%) [2,3]. Possible sources of infestation include hematogenous dissemination, local spread via pancreatobiliary ducts, and peripancreatic lymphatic invasion [1,2].

## Case presentation

A 27-year-old woman presented to our hospital with a progressing right hypochondral pain and cholestasis of 2 months duration associated with vomitis, pruritus and weight loss. Her personal and medical history was unremarkable. Physical examination revealed deep jaundice and scratch marks all over the body; there was a hard epigastric palpable mass with mild tenderness and no hepatomegaly.

Laboratory examination showed a serum bilirubin level of 88 mg/L (reference range 3-14 mg/L); aspartate aminotransferase, 147 U/L (reference range 0-46 U/L); alanine aminotransferase, 92 U/L (reference range 0-46 U/L); alkaline transferase, 488 U/L (reference range 64-300 U/L); and glutamyl transpeptidase 51 U/L (reference range 0-38 U/L). The hemoglobin, leukocytes and wafer values were normal.

Hydatid serology performed by an enzyme-linked immunosorbent assay was positive.

**Figure 1:**
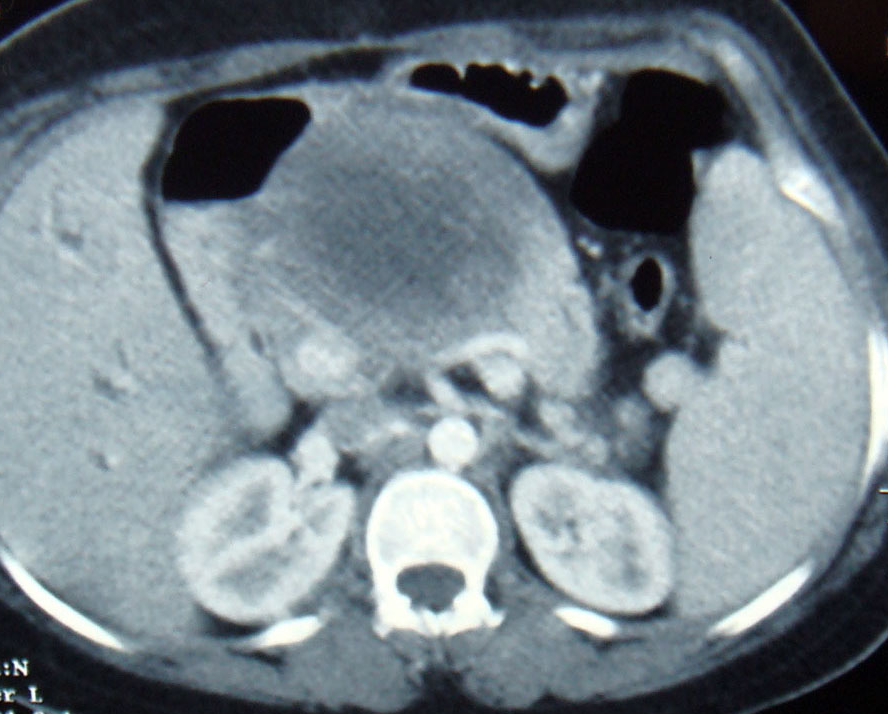
**Abdominal CT scan revealing a cystic tumor of the pancreatic head and body**.

Abdominal ultrasonography and CT scan revealed a 12 cm cystic tumor of the pancreatic head and body with a 10 mm wall thickness (Figure 1). The hepatic pedicule was compressed by this cyst resulting in a dilatation of the intraheapatic bile ducts and a segmental portal hypertension with 15 cm splenomegaly. No liver nodules, lymphadenopathy or ascites was observed.

**Figure 2:**
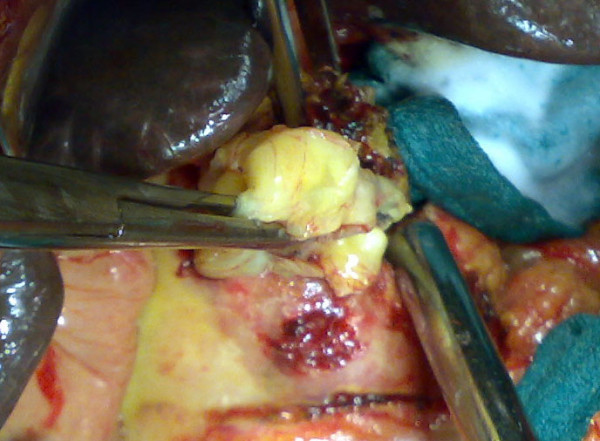
**Extraction of hydatic membranes after the opening of the pancreatic cyst**.

Laparotomy was performed finding a big cystic mass causing an important inflammation with extensive adherence to the épiploon, the inferior hepatic side and seeming after dissection to be continoued with the pancreatic tail (Figure 2). After protecting the operative area by a scolicidal solution, the cyst was opened. It's content was a transparent fluid with germinative and hydatid membranes. After cyst content removal, a partial cystectomy was performed and no communication was found between the cyst and the pancreatic duct or the biliary duct. An omentoplasty was done and the cystic cavity was drained. The presence of scolex in the cystic fluid tested positive. The postoperative period was uneventful. The daily drained liquid contained no amylase and no lipase. It's quantity rised till 1200 ml to progressively decrease. The drain was removed and the patient discharged on the twentyth postoperative day. The patient was free of symptoms and followed up after 15 month without any other abdominal localizations. The spleen size decreased and the doppler showed no portal hypertension.

## Discussion

As it is for pancreatic masses, the clinical presentation is variable and insidious, depending on the location and the size of the cyst [3,4,9]. Epigastric pain, discomfort, vomiting and weight loss are the main clinical symptoms [3-5]. However, complications could reveal this disease [2-4,10], such as acute pancreatitis, angiocholitis, cyst rupture into the peritoneal cavity, gastrointestinal tract abscess formation, episodes of anaphylactic shock or portal hypertension due to splenic vein or porta hepatis compression such as in our patient. The jaundice could be caused by its obstruction by hydatid material secondary to a cyst fistulisation or by the extrinsic compression of the common bile duct [2,8,9]. such as in our patient. Corpus and tail cysts rarely cause any symptoms.

The diagnosis of pancreatic cystic lesion may be performed by ultrasonography, CT scan or MRI [3].

In patients living in endemic areas, the presence of calcifications and hyperechoic band corresponding to daughter cysts or association with another abdominal localization leads to suspect the diagnosis of hydatid disease [3-5]. The enzyme-linked immunoadsorbent assay (ELIZA) test for echinococcal antigens, which is positive in over 85% of infected patients [4]. is also helpfull. Eventhough, the diagnosis of a hydatid cyst of the pancreas, which may easily be confused with other more commonly encountered cystic lesions, is extremely difficult and can rarely be established preoperatively [2,4].

Surgery remains the treatment of choice in hydatid disease, and to prevent parasitic dissemination, the protection of the operative site and sterilization of the cyst with a scolicidal solution such as hypertonic (20%) saline solution is necessary [2-4].

Many surgical techniques are available to remove the cyst. We used the pericystectomy with drainage of the residual cavity which might be the procedure of choice if the cyst is not related to the pancreatic duct [2,3,9]. If there is a connection with the duct, cystogastrostomy might be the procédure of choice. Partial pericystectomy or the insertion of a stent into the duct during surgery could also be the choice in some cases with a high risk of pancreatic fistulatotal [2,3]. For a hydatid cyst localized in the tail of the pancreas, the treatment of choice is a distal pancreatectomy with splenic conservation [3,4,9]. Combined to medical treatment using albendazole, the percutaneous drainage [3] and the laparoscopic resection of the cyst have also been reported [10].

## Conclusion

The primary hydatid cyst of the pancreas may be a causative factor for obstructive jaundice and portal hypertension. It should be considered in the differential diagnosis of all cystic lesions of the pancreas, especially in endemic areas.

## Consent

Written informed consent was obtained from the patient for publication of this case report and accompanying images.

## Competing interests

The authors declare that they have no competing interests.

## Authors' contributions

AO, KA and KM operated on the patient. HE took the photos. KHI participated in following up. All authors participated in writing the case report and revising the draft.
